# EPIRETINAL PROLIFERATION EMBEDDING WITHOUT INTERNAL LIMITING MEMBRANE PEELING FOR LAMELLAR MACULAR HOLE

**DOI:** 10.1097/IAE.0000000000004792

**Published:** 2026-01-30

**Authors:** Ginevra G. Adamo, Marco Pellegrini, Pietro M. Talli, Riccardo Mondin, Antonio Cartabellotta, Giuseppe Giannaccare, Marco Mura

**Affiliations:** * Department of Translational Medicine, University of Ferrara, Ferrara, Italy;; † Sant'Anna University Hospital, Ferrara, Italy;; ‡ Eye Clinic, Department of Surgical Sciences, University of Cagliari, Cagliari, Italy; and; § King Khaled Eye Specialist Hospital, Riyadh, Saudi Arabia.

**Keywords:** lamellar macular hole, epiretinal proliferation, ILM peeling, embedding technique, ellipsoid zone, macular surgery

## Abstract

**Purpose:**

To report anatomical and functional outcomes of epiretinal proliferation (EP) embedding without internal limiting membrane peeling for lamellar macular holes (LMHs) with EP.

**Methods:**

This retrospective interventional case series involved patients with idiopathic LMHs and EP who underwent pars plana vitrectomy using the EP-embedding technique without internal limiting membrane peeling. The EP was peeled centripetally, left connected to the LMH edge, and embedded into the retinal cleavage plane. 20% sulfur hexafluoride gas tamponade was applied. After surgery, patients were recommended to avoid lying face-up.

**Results:**

Fifteen eyes of 15 patients were included, and the mean follow-up was 12 ± 8 months. Lamellar macular hole closure was achieved in 100% of cases. Best-corrected visual acuity and central retinal thickness showed significant postoperative improvement, from 0.31 ± 0.20 logMAR (20/40 Snellen) and 156.5 ± 40.7 *µ*m preoperatively to 0.10 ± 0.12 logMAR (20/25 Snellen) (*P* = 0.002) and 221.5 ± 46.5 *µ*m (*P* < 0.001), respectively. Ellipsoid zone (EZ) and external limiting membrane restoration rates were 50% and 100%, respectively. No cases of full-thickness macular hole formation, LMH recurrence, or major complications were observed.

**Conclusion:**

EP-embedding without internal limiting membrane peeling yields favorable anatomical and functional outcomes in LMHs with EP and supports the nontractional pathophysiology of the condition.

## Abstract 2

Supplemental Digital Content is Available in the Text.

Preservation of the internal limiting membrane combined with embedding epiretinal proliferation may improve anatomical stability and functional recovery in lamellar macular holes, reflecting their nontractional nature. The authors report clinical outcomes from patients treated with this conservative surgical approach.

Epiretinal proliferation (EP) tissue associated with lamellar macular holes (LMHs) was first described in 2006 by Witkin et al^1^ as an “highly-reflective thick line with moderately reflective material filling the space between the retinal nerve fiber layer and the inner border of the epiretinal membrane” on ultra-high resolution optical coherence tomography (OCT). Pang et al^2^ later introduced the term “lamellar hole-associated epiretinal proliferation” (LHEP) to name this layer as it was believed to be associated only with LMH. Afterward, histopathology studies showed the fibroblasts and hyalocytes to be predominant in LHEP, whereas myofibroblasts represented the main component in conventional macular pseudoholes with epiretinal membranes (ERMs). Moreover, cells within LHEP seemed to possess little or no contractile properties in contrast to the conventional ERMs, highlighting differences in pathogenesis that could explain distinct disease progression and response to surgery in these cases.^3^ Based on these findings, Govetto et al^4^ proposed a new classification of LMH into tractional and degenerative types and reported the 95.8% of the degenerative LMHs to be characterized by the presence of EP. However, reports that followed demonstrated that the detection of this EP is not limited to the LMH, indeed it may also be present in ERM, full-thickness macular holes, and posterior uveitis.^5^ Recently, a team of experts in vitreoretinal diseases gathered and assessed published data on morphologic features of nonfull-thickness macular defect definition to establish a unified diagnostic approach based on OCT and to promote a standardized terminology for both clinical and research settings. Three conditions were identified: LMH, macular pseudohole, and ERM foveoschisis. They also decided to remove the “lamellar-macular hole associated” from the acronym LHEP because it was no longer correct, and to keep the remaining “EP” part for future descriptions.^6^

Several surgical outcomes have been reported for LMHs associated with EP, but it still remains controversial whether surgery is indicated for LMH associated with EP and, if surgery is performed, which surgical technique is most appropriate.

The purpose of this study was to investigate the visual and anatomical outcomes of a modified surgical technique for LMHs with EP for whom we performed the EP-embedding technique without internal limiting membrane (ILM) peeling.

## Methods

This retrospective interventional study enrolled consecutive patients who received surgical intervention for idiopathic LMH with EP. Treatments and follow-ups were conducted at the Ophthalmology Unit of Sant’Anna University Hospital (Ferrara, Italy) between March 2022 and March 2024. All patients provided written informed consent. The study adhered to the ethical standards of the 2013 Declaration of Helsinki and obtained approval from the local Institutional Review Board.

The classification of LMH was based on the criteria from the international panel of vitreoretinal experts: LMH was defined as an irregular foveal contour, foveal cavity with undermined edges, and at least one additional indicator of foveal tissue loss, in addition to the EP.^6^

Only patients who were followed up for at least 6 months postoperatively were included. All patients complained of visual acuity loss and/or metamorphopsia. Exclusion criteria were choroidal neovascularization, diabetic retinopathy, high myopia, other vascular retinal diseases, retinal dystrophies, glaucoma, uveitis and intraocular infections, trauma, any additional conditions potentially affecting best-corrected visual acuity (BCVA), excluding for lens opacity, and any prior intraocular surgeries other than uncomplicated cataract surgery.

**Fig. 1.**
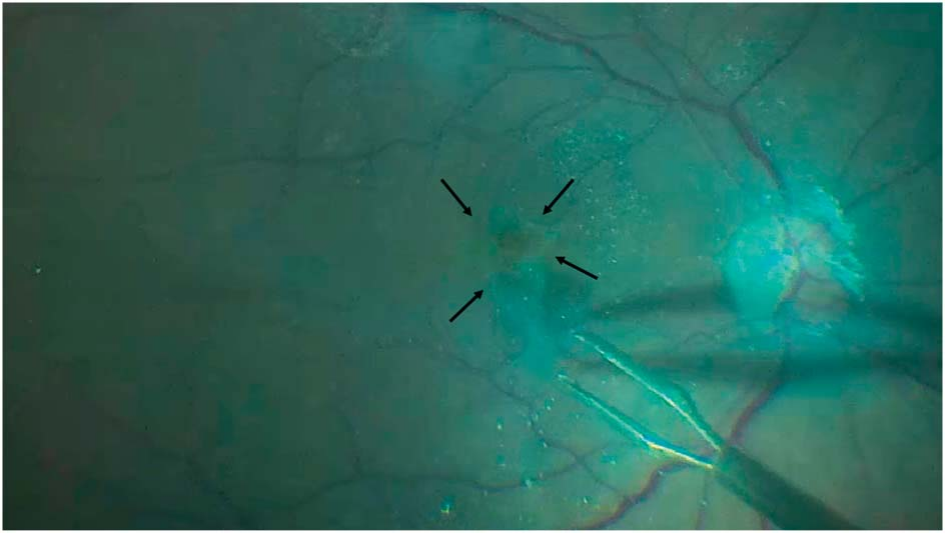
Intraoperative photograph showing the EP embedding surgical technique without ILM peeling for LMH. EP is centripetally peeled off from the retina and left attached to the edge of the LMH, the raised LHEP flap is visible (black arrows).

All patients received a complete ophthalmologic examination at baseline and at each postoperative visit, including measurement of BCVA, biomicroscopic examination, applanation tonometry, dilated fundoscopy, and macular OCT (Heidelberg Engineering GmbH, Heidelberg, Germany). All cases received 25-gauge transconjunctival, sutureless vitrectomy. Phakic patients older than 50 years underwent combined cataract surgery. After core vitrectomy and removal of the posterior hyaloid, triamcinolone acetonide was administered, followed by peripheral vitrectomy and vitreous base shaving. Membrane Blue-Dual (D.O.R.C., the Netherlands) was injected, and the EP was carefully peeled centripetally and repositioned over the fovea LMH using vitreoretinal forceps and left connected to the edges of the LMH without any further maneuvers (Figure 1). If the EP exceed the size of the LMH area, it was trimmed using the vitreous cutter. No ILM peeling was performed. A fluid–air exchange was performed, and at the end of the procedure, the vitreous cavity was filled with 20% sulfur hexafluoride gas (see **Video**, **Supplemental Digital Content 1**, http://links.lww.com/IAE/C830, which highlights the key steps of the technique). Postoperatively, patients were recommended to avoid lying face-up, and face-down positioning was not maintained.

The main outcome measures were BCVA, central retinal thickness, continuity of the external limiting membrane (ELM), and continuity of the ellipsoid zone (EZ). The EZ was defined as restored when continuous EZ integrity was assessed at the fovea after surgery.

All data were recorded in an online database, and statistical analysis was performed using SPSS (Statistical Package for Social Sciences; IBM corp. Armonk, NY). Values are expressed as mean ± SD for continuous variables and as individual counts and percentages for categorical values. Snellen BCVA was converted to logMAR for statistical purposes. The nonparametric Wilcoxon test was used to compare pre- and postoperative changes in BCVA and central retinal thickness. The McNemar test was used to compare pre- and postoperative disruption of ELM and EZ. *P* values of less than 0.05 were considered to be statistically significant.

## Results

A total of 15 patients underwent surgery using the EP-embedding technique without ILM peeling. Preoperative and postoperative characteristic of all 15 patients are summarized in Table 1. The mean age was 72.73 ± 11.73 (range, 49–89 years); eight patients (53%) were male, and seven patients (47%) were female. At presentation, six of 15 eyes (40%) were pseudophakic. All nine phakic patients received concurrent phacoemulsification combined with intraocular lens implantation. The mean postoperative follow-up period was 12.33 ± 8.40 months (range, 6–30 months).

**Table 1.** Pre- and Postoperative Characteristics of All Patients Included

Case	Age (years)	Gender	Lens status	BCVA LogMAR (Snellen)		CRT (μm)		ELM Integrity		EZ Integrity			
No.				Preop	Postop	Preop	Postop	Preop (Length, μm)	Postop	Preop (Length, μm)	Postop (Length, μm)			Pre-op Foveal Bump	Follow-Up (months)
1	70	F	Phakic	0 (20/20)	0.02 (20/21)	225	256	Intact	Intact	Intact	Intact	Not observed	6
2	89	M	Pseudophakic	0.22 (20/33)	0.13 (20/27)	118	159	Intact	Intact	Disrupted (142)	Intact	Not observed	6
3	81	F	Pseudophakic	0.19 (20/31)	0.08 (20/24)	149	185	Intact	Intact	Intact	Intact	Not observed	8
4	80	F	Phakic	0.34 (20/44)	0.13 (20/27)	179	232	Intact	Intact	Intact	Intact	Observed	18
5	71	F	Phakic	0.53 (20/74)	0.02 (20/21)	206	279	Intact	Intact	Intact	Intact	Observed	6
6	66	F	Phakic	0.13 (20/27)	0 (20/20)	164	218	Disrupted (82)	Intact	Disrupted (163)	Intact	Observed	8
7	75	M	Pseudophakic	0.4 (20/50)	0.02 (20/21)	124	164	Intact	Intact	Disrupted (149)	Intact	Observed	30
8	75	M	Phakic	0.13 (20/27)	0.02 (20/21)	110	198	Intact	Intact	Disrupted (171)	Disrupted (336)	Observed	15
9	49	M	Pseudophakic	0.3 (20/40)	0.13 (20/27)	171	281	Intact	Intact	Intact	Intact	Not observed	8
10	51	M	Phakic	0.02 (20/21)	0.02 (20/21)	171	298	Intact	Intact	Intact	Intact	Observed	6
11	62	F	Phakic	0.34 (20/44)	0 (20/20)	225	232	Intact	Intact	Intact	Intact	Observed	12
12	73	M	Phakic	0.53 (20/68)	0.1 (20/25)	164	221	Intact	Intact	Intact	Intact	Not observed	6
13	85	M	Pseudophakic	0.3 (20/40)	0.3 (20/40)	110	146	Intact	Intact	Disrupted (141)	Disrupted (129)	Observed	22
14	82	M	Pseudophakic	0.53 (20/68)	0.4 (20/50)	125	252	Intact	Intact	Intact	Disrupted (202)	Not observed	6
15	82	F	Phakic	0.7 (20/100)	0.16 (20/29)	107	202	Intact	Intact	Disrupted (189)	Disrupted (167)	Not observed	28

CRT, central retinal thickness.

Lamellar macular hole closure was obtained in 100% of cases. The mean BCVA significantly improved over the course of the study, from 0.31 ± 0.20 logMAR (20/40 Snellen) preoperatively to 0.10 ± 0.12 logMAR (20/25 Snellen) at the final postoperative visit (*P* = 0.002, Wilcoxon signed-rank test). The mean central retinal thickness also significantly increased from 156.53 ± 40.74 *µ*m preoperatively to 221.53 ± 46.47 *µ*m postoperatively (*P* < 0.001, Wilcoxon signed-rank test).

**Fig. 2.**
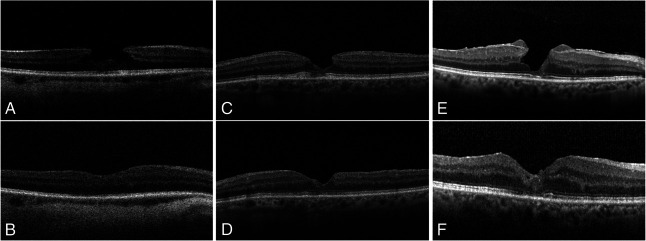
Pre- and postoperative OCT images. Case 5 A LMH with continuous EZ, B at 6 months postsurgery with covered foveal cleavage and continuous EZ. Case 2 C LMH with disrupted EZ, D at 6 months postsurgery showing covered foveal cleavage and restored EZ. Case 8 E LMH with discontinuous EZ, F at 15 months postsurgery with covered foveal cleavage and disrupted EZ.

Preoperatively, ELM disruption was observed in one eye (6.67%) and showed complete postoperative restoration. Ellipsoid zone disruption was detected in six eyes (40%) before surgery and was quantified by measuring the widest linear extent of disruption identified on OCT scans, with a mean preoperative extent of 159.2 ± 18,8 *µ*m. Postoperatively, EZ disruption resolved in three eyes presenting EZ disruption, whereas one eye with an intact preoperative EZ developed a new disruption at follow-up. Overall, EZ disruption was present in four eyes (26.67%) postoperatively (*P* = 0.625, McNemar test) (Figure 2), with a mean postoperative extent of 208.5 ± 90.1 *µ*m. In addition, a preoperative foveal bump was observed in eight patients (53.3%).

No intraoperative or postoperative complications were observed, including the occurrence of a FTMH, recurrence of LMH, LMH with EP, or ERM. A transient increase in intraocular pressure was detected in three patients (20%), which was successfully managed with topical therapy, without the need for additional interventions.

## Discussion

In this study, we evaluated the visual and anatomical outcomes after a modified surgical approach, EP-embedding without ILM peeling, in patients with LMHs associated with EP and investigated the postoperative course.

The presence of EP poses unique surgical challenges and may affect anatomical and functional outcomes. While previous studies have reported variable results after surgery for LMHs with EP, some authors have demonstrated encouraging visual and anatomical improvements. For instance, Parisi et al and Murphy et al reported favorable outcomes, and Lai et al showed comparable functional outcomes in both LMHs with and without EP.^7–9^ Conversely, other studies reported worse outcomes, supporting the hypothesis of a degenerative pathology.^10–14^

In recent years, various alternative surgical techniques have been proposed to enhance treatment outcomes. Increasing evidence supports the idea that the preservation of EP may be a key factor for achieving successful results, regardless of the surgical technique used.^9,15–18^

Nevertheless, indication for ILM peeling in these cases remains controversial, as it has been reported that the involvement of retinal traction in LMHs with EP is limited. The therapeutic benefit of ILM removal in LMHs with EP was observed to be less evident compared with tractional LMHs or macular pseudoholes.^19,20^ Furthermore, Hirano et al^21^ observed that en face OCT imaging of both tractional LMHs and macular pseudoholes revealed retinal folds, whereas no retinal folds were observed in LMHs with EP.

In addition, several studies reported the ILM peeling to be associated with iatrogenic damage to the inner retinal structures, including mechanical trauma to Müller cells, disruption of the inner retina layers, and thinning of the ganglion cell layer.^22^ Postoperative OCT frequently reveals inner retinal thinning, dissociated optic nerve fiber layer patterns, reduced retinal sensitivity, and microscotomas, which may compromise visual function.^23^ Furthermore, conventional membrane peeling in LMHs defined as degenerative has been linked to postoperative complications, such as the formation of FTMHs and macular thinning.^12,13,24,25^

Our results suggest that vitrectomy and EP embedding alone, with intentional omission of ILM peeling, can achieve effective therapeutic outcomes in the treatment of LMHs as the compensation for the inner foveal defect may be more critical than relieving tractions. Epiretinal proliferation is a viscous, elastic substance with has a consistency resembling that of a chewing gum. In most cases, EP does not stain with blue dyes. Moreover, during peeling, the separation between EP and the ILM occurs more easily, as adhesion to the ILM is almost absent, reducing the risk of retinal damage. Indeed, after peeling the EP, it is possible to stain with Membrane Dual Blue and identify the intact ILM. In fact, unlike epiretinal membranes, it is uncommon for the ILM to be removed together with the EP.

In our cohort, postoperatively, the ELM continuity was restored in 100% of eyes (1/1), and restoration of the EZ was observed in 50% of previously disrupted cases (3/6). We observed both restoration of foveal contour and improvement in BCVA. In addition, no cases of LMH recurrences were observed, contradicting the expectation for a progressive worsening that is typical of a truly degenerative condition. These findings demonstrate considerable healing capability, with significant functional and microstructural improvements after surgical treatment, suggesting that “degenerative” LMHs may possess regenerative potentials. This challenges the traditional view of degenerative LMHs as irreversible conditions.

In support of this idea, EP has been suggested to result from the proliferation of Müller cells within the middle retinal layers at sites of retinal defects during the development of LMHs.^2^ This hypothesis is in accordance to other studies demonstrating the presence of larger and deeper retinal defects, along with an higher incidence of inner segment/outer segment or ellipsoid disruption, in LMHs with EP compared with LMHs without EP.^2,12^ Morphological and functional differences suggest that LMHs with EP may have different etiologies than those not associated with EP, therefore potentially requiring different treatments.^26^

Our results align with recent evidences from Pertile et al,^27^ who reported reconstitution of the outer retinal layers and a reduction in autofluorescence abnormalities after surgical intervention for both LMHs with and without EP, and ERM foveoschisis. Furthermore, spontaneous closure of degenerative LMHs associated with EP has been documented, further questioning their classification as purely degenerative entities. Although rarely reported, the spontaneous healing process implies an active reparative mechanism, supporting that these lesions are not strictly degenerative.^28^

Despite these encouraging results, our study presents limitations. The relatively small sample size and the variable duration of follow-up limit the generalizability of our findings. In addition, the absence of a control group undergoing conventional peeling techniques limits direct comparative analysis. Moreover, in our consecutively enrolled cohort, nine of 15 patients did not exhibit preoperative alterations of the outer retinal layers. More advanced disease, characterized by extensive retinal damage, may be associated with less favorable surgical outcomes; accordingly, studies stratifying surgical results by the degree of outer retinal degeneration are warranted. Furthermore, in this study, EZ and ELM disruption were quantified by measuring the maximum linear extent of interruption observed on OCT scans; however, a more comprehensive and representative assessment might be achieved by quantifying the areas of EZ and ELM disruption. Finally, further prospective, randomized controlled studies involving larger cohorts and extended follow-up periods are necessary to assess the long-term functional and anatomical advantages of this surgical approach.

To conclude, the study supports the concept that LMH associated with EP constitutes a distinct clinical and pathological entity that benefits from an individualized therapeutic approach. The EP-embedding technique, performed without peeling of ILM, may represent an effective and minimally invasive approach for both morphologic and functional improvements.

Avoiding the ILM peeling in this surgical approach is consistent with the unique pathophysiology of LMHs associated with EP, characterized by minimal or absent vitreoretinal traction as evidenced by structural imaging and clinical finding. Consequently, the conventional rationale for ILM removal is less relevant. In addition, the potential adverse effects of ILM peeling warrant a careful consideration. Collectively, these factors suggest that ILM peeling in LMHs with EP may confer limited therapeutic benefit while posing significant risk to retinal integrity and function. Our results, aligned with current literature, support that ILM preservation combined with EP embedding may enhance anatomical stability and functional recovery, consistent with the nontractional nature of this pathology and advocating a more conservative surgical approach.

## Supplementary Material

## References

[1] Witkin AJ Ko TH Fujimoto JG Redefining lamellar holes and the vitreomacular interface: an ultrahigh-resolution optical coherence tomography study. Ophthalmology 2006;113:388–397. 16513456 10.1016/j.ophtha.2005.10.047 PMC1940046

[2] Pang CE Spaide RF Freund KB. Epiretinal proliferation seen in association with lamellar macular holes: a distinct clinical entity. Retina 2014;34:1513–1523. 24732699 10.1097/IAE.0000000000000163

[3] Compera D Entchev E Haritoglou C Lamellar hole-associated epiretinal proliferation in comparison to epiretinal membranes of macular pseudoholes. Am J Ophthalmol 2015;160:373–384.e1. 25982970 10.1016/j.ajo.2015.05.010

[4] Govetto A Dacquay Y Farajzadeh M Lamellar macular hole: two distinct clinical entities?. Am J Ophthalmol 2016;164:99–109. 26898164 10.1016/j.ajo.2016.02.008

[5] Itoh Y Levison AL Kaiser PK Prevalence and characteristics of hyporeflective preretinal tissue in vitreomacular interface disorders. Br J Ophthalmol 2016;100:399–404. 26206790 10.1136/bjophthalmol-2015-306986 PMC4936527

[6] Hubschman JP Govetto A Spaide RF Optical coherence tomography-based consensus definition for lamellar macular hole. Br J Ophthalmol 2020;104:1741–1747. 32107208 10.1136/bjophthalmol-2019-315432

[7] Parisi G Fallico M Maugeri A Primary vitrectomy for degenerative and tractional lamellar macular holes: a systematic review and meta-analysis. PLoS One 2021;16:e0246667. 33667237 10.1371/journal.pone.0246667 PMC7935291

[8] Murphy D Rees J Steel D. Surgical interventions for lamellar macular holes. Cochrane Database Syst Rev 2021;11:CD013678. 34748208 10.1002/14651858.CD013678.pub2 PMC8574711

[9] Lai TT Chen SN Yang CM. Epiretinal proliferation in lamellar macular holes and full-thickness macular holes: clinical and surgical findings. Graefe’s Arch Clin Exp Ophthalmol 2016;254:629–638. 26311257 10.1007/s00417-015-3133-9

[10] Coassin M Mastrofilippo V Stewart JM Lamellar macular holes: surgical outcome of 106 patients with long-term followup. Graefe’s Arch Clin Exp Ophthalmol 2018;256:1265–1273. 29785511 10.1007/s00417-018-3989-6

[11] Sanisoglu H Elbay A Sevim S Surgical therapy versus observation for lamellar macular hole: a retrospective comparison study. Clin Ophthalmol 2013;7:1843–1848. 24092963 10.2147/OPTH.S46283 PMC3788682

[12] Ko J Kim GA Lee SC Surgical outcomes of lamellar macular holes with and without lamellar hole-associated epiretinal proliferation. Acta Ophthalmologica 2017;95:e221–e226. 27647708 10.1111/aos.13245

[13] Choi WS Merlau DJ Chang S. Vitrectomy for macular disorders associated with lamellar macular hole epiretinal proliferation. Retina 2018;38:664–669. 28301339 10.1097/IAE.0000000000001591

[14] Schumann RG Compera D Schaumberger MM Epiretinal membrane characteristics correlate with photoreceptor layer defects in lamellar macular holes and macular pseudoholes. Retina 2015;35:727–735. 25341885 10.1097/IAE.0000000000000375

[15] Yang YS Lee JS Son G Sohn J. Epiretinal proliferation associated with lamellar hole or macular hole: origin and surgical prognosis. Korean J Ophthalmol 2019;33:142–149. 30977324 10.3341/kjo.2018.0070 PMC6462480

[16] Shiode Y Morizane Y Takahashi K Embedding of lamellar hole-associated epiretinal proliferation combined with internal limiting membrane inversion for the treatment of lamellar macular hole: a case report. BMC Ophthalmol 2018;18:257. 30249209 10.1186/s12886-018-0926-8 PMC6154401

[17] Takahashi K Morizane Y Kimura S Results of lamellar macular hole-associated epiretinal proliferation embedding technique for the treatment of degenerative lamellar macular hole. Graefe’s Arch Clin Exp Ophthalmol 2019;257:2147–2154. 31342148 10.1007/s00417-019-04425-9

[18] Ho TC Ho AY Chen MS. Reconstructing foveola by foveolar internal limiting membrane non-peeling and tissue repositioning for lamellar hole-related epiretinal proliferation. Sci Rep 2019;9:16030. 31690760 10.1038/s41598-019-52447-4 PMC6831694

[19] Purtskhvanidze K Balken L Hamann T Long-term follow-up of lamellar macular holes and pseudoholes over at least 5 years. Graefes Arch Clin Exp Ophthalmol 2018;256:1067–1078. 29623462 10.1007/s00417-018-3972-2

[20] Figueroa MS Govetto A Steel DH Pars plana vitrectomy for the treatment of tractional and degenerative lamellar macular holes: functional and anatomical results. Retina 2019;39:2090–2098. 30312255 10.1097/IAE.0000000000002326

[21] Hirano M Morizane Y Kimura S Assessment of lamellar macular hole and macular pseudohole with a combination of en face and radial B-scan optical coherence tomography imaging. Am J Ophthalmol 2018;188:29–40. 29360459 10.1016/j.ajo.2018.01.016

[22] Murphy DC Fostier W Rees J Steel DH. Foveal sparing internal limiting membrane peeling for idiopathic macular holes: effects on anatomical restoration of the fovea and visual function. Retina 2020;40:2127–2133. 31860521 10.1097/IAE.0000000000002724

[23] Sadeghi E Colorado-Zavala MF Almuhtaseb H Anatomical and functional changes after internal limiting membrane peeling. Surv Ophthalmol 2025;70:357–368. 39842613 10.1016/j.survophthal.2025.01.008

[24] Parolini B Schumann RG Cereda MG Lamellar macular hole: a clinicopathologic correlation of surgically excised epiretinal membranes. Invest Ophthalmol Vis Sci 2011;52:9074–9083. 22025575 10.1167/iovs.11-8227

[25] Pang CE Maberley DA Freund KB Lamellar hole-associated epiretinal proliferation: a clinicopathologic correlation. Retina 2016;36:1408–1412. 27164549 10.1097/IAE.0000000000001069

[26] Pang CE Spaide RF Freund KB. Comparing functional and morphologic characteristics of lamellar macular holes with and without lamellar hole-associated epiretinal proliferation. Retina 2015;35:720–726. 25521439 10.1097/IAE.0000000000000390

[27] Pertile G Iacovello D Maraone G Lamellar macular defects: are degenerative lamellar macular holes truly degenerative?. Front Med (Lausanne) 2023;10:1156410. 37138761 10.3389/fmed.2023.1156410 PMC10149835

[28] Preti RC Zacharias LC Cunha LP Spontaneous closure of degenerative lamellar macular hole with epiretinal membrane proliferation. Int J Retina Vitreous 2021;7;(1):64. 34702375 10.1186/s40942-021-00339-z PMC8549372

